# Structural-color meta-nanoprinting embedding multi-domain spatial light field information

**DOI:** 10.1515/nanoph-2024-0019

**Published:** 2024-03-06

**Authors:** Congling Liang, Jiahao Wang, Tian Huang, Qi Dai, Zile Li, Shaohua Yu, Gongfa Li, Guoxing Zheng

**Affiliations:** Electronic Information School, and School of Microelectronics, 12390 Wuhan University, Wuhan, 430072, China; Peng Cheng Laboratory, Shenzhen, 518055, China; Wuhan Institute of Quantum Technology, Wuhan, 430206, China; Suzhou Institute of Wuhan University, Suzhou, 215123, China; Key Laboratory of Metallurgical Equipment and Control Technology of Ministry of Education, 47900 Wuhan University of Science and Technology, Wuhan, 430081, China

**Keywords:** metasurface, meta-nanoprinting, holography

## Abstract

Recently, multifunctional metasurface has showcased its powerful functionality to integrate nanoprinting and holography, and display ultracompact meta-images in near- and far-field simultaneously. Herein, we propose a tri-channel metasurface which can further extend the meta-imaging ranges, with three independent images located at the interface, Fresnel and Fourier domains, respectively. Specifically, a structural-color nanoprinting image is decoded right at the interface of the metasurface, enabled by varying the dimensions of nanostructures; a Fresnel holographic image and another Fourier holographic image are present at the Fresnel and Fourier (far-field) domains, respectively, enabled by geometric phase. The spectral and phase manipulation capabilities of nanostructures have been maximized, and the spatial multiplexing capabilities for diffraction in metasurfaces have also been fully exploited. By leveraging the design freedom enabled through the tuning of the geometric size and orientation of nanostructures, as well as optimizing the diffraction spatial light wave transformation, the encoding of multiple images on the single-celled metasurface is achieved. More interestingly, due to the spatial separation of images across different channels, crosstalk is virtually eliminated, effectively enhancing imaging quality. The proposed metasurface offers several advantages, including a compact design, easiness of fabrication, minimal crosstalk, and high storage density. Consequently, it holds promising applications in image display, data storage, information encryption, anti-counterfeiting, and various other fields.

## 1 Introduction

Constructed with subwavelength-scaled unit cells, metasurfaces showcase a remarkable capacity for light manipulation, paving the way for a revolution in the planarization of optical components [1], [2], [3], [4]. Metasurfaces find extensive applications across a broad spectrum, encompassing imaging [5], [6], [7], [8], [9], [10], optical computing [11], [12], [13], [14], [15], [16], image display [17], [18], [19], [20], [21], [22], anti-counterfeiting [23], [24], [25], encryption [26], [27], [28], [29], [30], [31], [32], [33], *etc*. Significantly, meta-image display and optical storage emerge as two prominent domains where metasurface have made noteworthy progress [19], [20], [25], [34], [35]. Meta-nanoprinting and meta-holography are two main platforms to achieve meta-image display and storage. For meta-nanoprinting, the intensity or color of the image is modulated by the metasurface on a pixel-by-pixel basis. Meta-holography works on a diffraction basis: the amplitude and/or the phase of light are modulated by the metasurface.

To enhance storage capacity, various types of metasurfaces that integrate meta-nanoprinting and meta-holography have been proposed [23], [24], [25], [28], [32], [36], [37], [38], [39], [40], [41]. For instance, nanoprinting and holography can be designed to operate in a pair of orthogonally polarized light channels [36]. However, the image quality is sensitive to fabrication errors in both dimensions in the orthogonally-polarized direction. Alternatively, by in-plane interleaving and spatially stacking nanostructures that achieve phase and intensity/spectrum modulation, nanoprinting and holography can be simultaneously realized [24], [28], [37], [38]. However, interleaving can lead to degradation in image quality, and stacked multilayer structures can complicate the fabrication process. Therefore, a single-layered configuration with simultaneous modulation of multiple optical parameters is preferred for ease of fabrication and compactness. In order to integrate color nanoprinting and holography with a single-layered metasurface, the primary approach involves altering the dimensions of nanostructures to control the transmission and reflection spectra, simultaneously modifying the arrangement of the nanostructures for phase modulation [39], [40], [41]. Detour phase and geometric phase modulations are both employed to generate holographic images. In these methodologies, the holographic images are intended to be observed at a specific domain plane, limiting the benefits of holography for light field modulation in space. As for multiplanar meta-holography, issues such as crosstalk and noise are naturally unavoidable, which results in image degradation. In summary, there is a demand for metasurfaces with high information capacity, minimal crosstalk, simplified configurations, and high image quality.

**Figure 1:**
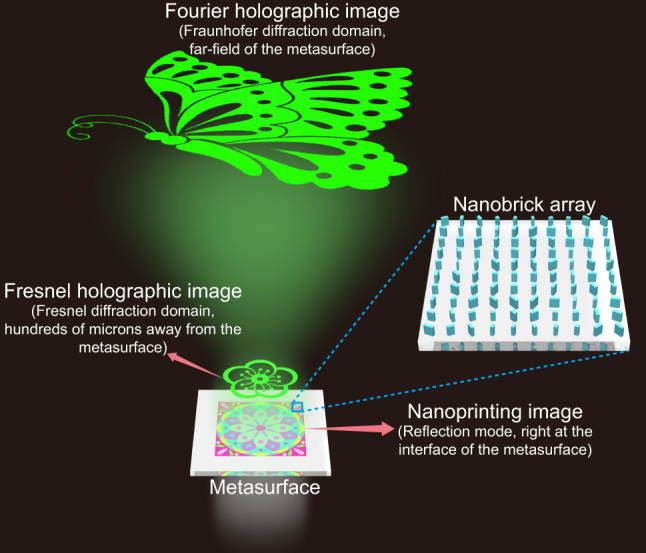
Schematic diagram of the tri-channel multifunctional metasurface. The metasurface is composed of nanobricks with varying geometric parameters and orientation angles. The colored nanoprinting image can be observed right at the metasurface plane (interface plane), while the Fresnel holographic image and the Fourier holographic image can be observed within specific diffraction domains.

To address these challenges, we present a new approach to record information with metasurfaces, with which color meta-nanoprinting is imbedded with multi-domain spatial light field information. The fundamental goal of this research is to boost storage density while upholding superior image quality. The distinctiveness of our approach lies in the development of functionality for spectral and phase modulation of the unit cells comprising the metasurface, as well as the utilization of the non-injective properties and the robustness of holography. Through this approach, we encoded three distinct images into a single metasurface, including a color nanoprinting image, a Fresnel holographic image, and a Fourier holographic image. What distinguishes this approach is the capacity to observe these images at planes positioned at specific distances relative to the metasurface, guided by predefined optical paths, as shown in Figure 1. The deliberate spatial separation of images across different channels in our design effectively eliminates the issue of crosstalk, thereby preserving the high quality of each individual image. Furthermore, the metasurface presented in our study is notable for its minimalist design, characterized by a single-celled and single-layered configuration that streamlines the fabrication process. The simplicity of design and easiness of production render the metasurface accessible, practical, and promising for a wide range of applications. With its compact design, easiness of fabrication, minimal crosstalk, and substantial storage density, the tri-channel metasurface presents exciting prospects in various fields, spanning from image display and data storage to information encryption, anti-counterfeiting measures, and beyond.

## 2 Design principle of the tri-channel multifunctional metasurface

The control of structural colors through Mie resonance in metasurfaces is a fascinating and intricate phenomenon. It relies on the manipulation of the interaction between incident light and resonant nanostructures. These structures are meticulously designed to exhibit Mie resonances at specific wavelengths, causing a strong scattering of light [20], [40], [41]. The reflected or transmitted spectra of the nanostructures can be modulated by adjusting their geometric parameters. Taking the total reflection spectrum of the anisotropic nanostructures into consideration, the reflectance is the average of the reflectance along its long and short axes. Consequently, the reflectance of an anisotropic nanostructure in an unpolarized light path remains invariant with respect to the orientation of the nanostructure. Therefore, the orientation angle of the nanostructures can be strategically engineered to manipulate the phase of light, leveraging the geometric phase, namely the Pancharatnam–Berry phase (PB phase), to generate holographic images at specific diffraction distances.

Based on this foundation, three distinct information channels can be established by utilizing the metasurface. Each nanostructure possesses the capability to manipulate the spectrum and phase of light. In Channel 1, the spectral modulation of the nanostructures under non-polarized illumination is harnessed. The theoretical model can be expressed as follows:

$$R\left(\lambda \right)=\frac{{\left|r_{l}\left(\lambda \right)\right|}^{2}+{\left|r_{s}\left(\lambda \right)\right|}^{2}}{2}$$

Here, ***r***_*l*_ and ***r***_*s*_ represent the complex reflection coefficients along the long and short axes of the nanostructure at wavelength *λ*, respectively, while *R*(*λ*) represents the reflectance at the same wavelength.

Considering the geometric phase, the orientation angle *θ*, which signifies the angle between the long axis of the nanobrick and the *x*-axis, becomes a crucial factor. In transmission mode, the complex amplitude ***E*** of the output light with an additional geometric phase can be expressed as:

$$E=\frac{t_{l}-t_{s}}{2}{\text{e}}^{\pm \text{i}2\theta }$$

In this equation, ***t***_*l*_ and ***t***_*s*_ denote the complex transmission coefficients along the long and short axes of the nanostructure at the design wavelength, while “+” and “−” correspond to left circular-handed polarized (LCP) and right circular-handed polarized (RCP) light, respectively. The phase of the complex value $\frac{t_{l}-t_{s}}{2}$ represents the propagation phase, which is related to the size of the nanoblock and independent of the orientation angle.

In Channel 2, the Fresnel diffraction formula is utilized:

(1) $$\begin{array}{ll} U\left(x,y\right) & =\frac{\exp\left(ikz\right)}{i\lambda z}\iint E\left(x_{1},y_{1}\right)\\ & \;\cdot \exp\left\{\frac{ik}{2z}\left[{\left(x-x_{1}\right)}^{2}+{\left(y-y_{1}\right)}^{2}\right]\right\}\text{d}x_{1}\text{d}y_{1} \end{array}$$

In Channel 3, the Fraunhofer diffraction formula is employed:

(2) $$\begin{array}{ll} U\left(x,y\right) & =\frac{\exp\left[ik\left(z+\frac{x^{2}+y^{2}}{z}\right)\right]}{i\lambda z}\iint E\left(x_{1},y_{1}\right)\\ & \;\cdot \exp\left[-\frac{ik}{z}\left(xx_{1}+yy_{1}\right)\right]\text{d}x_{1}\text{d}y_{1} \end{array}$$

wherein, $E\left(x_{1},y_{1}\right)$ represent the complex amplitude of the point with coordinates $\left(x_{1},y_{1}\right)$ on the metasurface, $U\left(x,y\right)$ represent the complex amplitude of the point with coordinates $\left(x,y\right)$ on the observation plane, *z* represents the diffraction distance, *λ* is the wavelength, and *k* is the wavenumber.

Fresnel holography operates in the near-field region, where the distance between the hologram and the observation plane is comparable to the size of the metasurface. The holographic image recorded in Fresnel hologram can be reconstructed using the convolution method, which can be viewed as the convolution of a linear space-invariant system and a transfer function. The distance between the hologram and the reconstruction plane affects the reconstructed image. On the other hand, Fourier holography operates in the far-field region, where the observation plane is placed at a considerable distance from the metasurface compared to its size. The holographic image recorded in Fourier hologram can be directly reconstructed by using Fourier-transformation, eliminating the necessity for angular spectrum decomposition. The reconstructed image in Fourier holography is not affected by changes in the distance between the hologram and the reconstruction plane. Therefore, the reconstructed image remains unchanged regardless of the distance. In summary, Fresnel holography and Fourier holography differ in their operational regions, reconstruction techniques, and dependence on the distance between the metasurface and the reconstruction plane. These characteristics enable the integration of both types of holography into a single metasurface.

**Figure 2:**
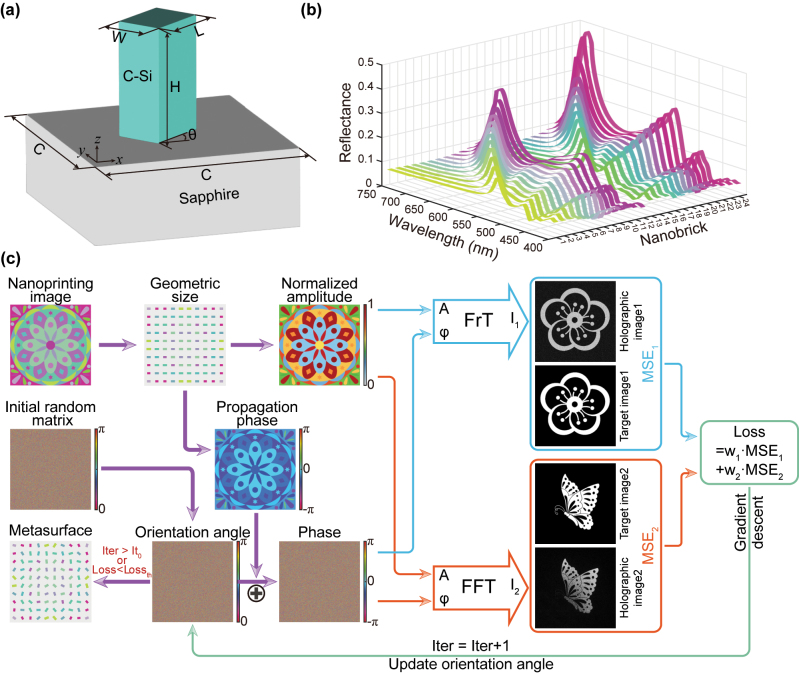
Metasurface design workflow. (a) Schematic diagram of the nanobrick unit-cell. (b) Simulated reflectance of nanobricks with varying geometric parameters, with the curve colors corresponding to simulated colors. (c) Flowchart outlining the design process of the metasurface. The length and width of the nanobricks are determined based on the color of the target nanoprinting image pixels. Then, amplitude distribution and propagation phase profile can be obtained. The holographic images corresponding to a specific orientation angle distribution are acquired through forward calculations of Fresnel transmission (FrT) and fast Fourier transform (FFT). The orientation angle is updated using the gradient descent algorithm, which relies on the loss function and gradient. After completing the iterations, the arrangement of the nanobricks is determined by combining geometric sizes with the orientation angles.

To construct the metasurface, electromagnetic simulations were initially performed to obtain the responses of nanostructures with different geometric parameters. As illustrated in Figure 2(a), the key parameters of the nanobrick include its length (*L*), width (*W*), height (*H*), and cell period (*C*). The height was kept constant at 230 nm in the simulation, corresponding to the top silicon thickness of an off-the-shelf Silicon-On-Sapphire (SOS) wafer. The cell periods were selected to be 400 nm × 400 nm. The length and width were systematically varied in 10 nm steps to investigate the spectral manipulation of the nanobrick, ranging from 90 to 200 nm in length and 60–70 nm in width, respectively. Utilizing the CST Studio Suite software, the spectral responses of nanostructures with different geometries were obtained, as depicted in Figure 2(b). The widths of nanobricks with serial numbers 1–12 and 13–24 are 60 nm and 70 nm, respectively. Maintaining a consistent width, the lengths of nanobricks increase from 90 nm to 200 nm as the serial numbers progress. The changes in geometry led to corresponding alterations in the spectral response curve, ultimately resulting in shifts in the structural color associated with the nanostructure. Specifically, as the long axis of the nanostructure increases, a red shift occurs in the reflection peak, resulting in a gradual change in the reflected light color toward red.

The metasurface design process primarily involves two main steps: determining the geometric parameters of the nanobricks and optimizing the orientation angle distribution, as illustrated in Figure 2(c). Based on the color information of each pixel in the nanoprinting image, the length and width of the nanobrick can be determined on a point-by-point basis. Accordingly, in accordance the results of electromagnetic simulations, the amplitude distribution and propagation phase distribution at the operating wavelength can be determined by the absolute value and argument value of the complex value (***t***_*l*_−***t***_*s*_)/2, respectively. The optimization process begins with randomly distributed orientation angles within the range of 0 to *π*. The phase distribution of the metasurface is determined by the combination of both geometric phase and propagation phase. Based on the complex amplitude distribution on the metasurface plane, the diffraction intensity on a specific plane can be calculated using Equations (1) and (2). Subsequently, the mean square error (MSE) between the holographic images and the target images can be computed. The loss function is defined as:

$$\text{Loss}=w_{1}\cdot {\text{MSE}}_{1}+w_{2}\cdot {\text{MSE}}_{2}$$

Here, *MSE*_1_ and *MSE*_2_ represent the MSE values of the two holographic channels, while *w*_1_ and *w*_2_ are the weight coefficients. Specifically, the ratio of *w*_1_ to *w*_2_ is inversely proportional to the ratio of the sum of pixel intensities in the two target images *I*_1_ and *I*_2_.

$$\frac{w_{1}}{w_{2}}=\frac{\sum I_{2}}{\sum I_{1}}$$

Upon obtaining the *Loss*, the gradient is calculated, and subsequently, the orientation angle distribution is updated based on this gradient. Afterwards, the diffraction images and loss function corresponding to the new orientation angle distribution are calculated. The optimization loop continues until either the specified iteration limit *It*_0_ is reached or the loss value falls below the set threshold *Loss*_th_. Once this condition is met, the optimization loop breaks, and the optimized orientation angle distribution is determined. The arrangement of the nanobrick arrays is finalized by combining geometric sizes with the distribution of orientation angles.

## 3 Experimental demonstration of the tri-channel metasurface

**Figure 3:**
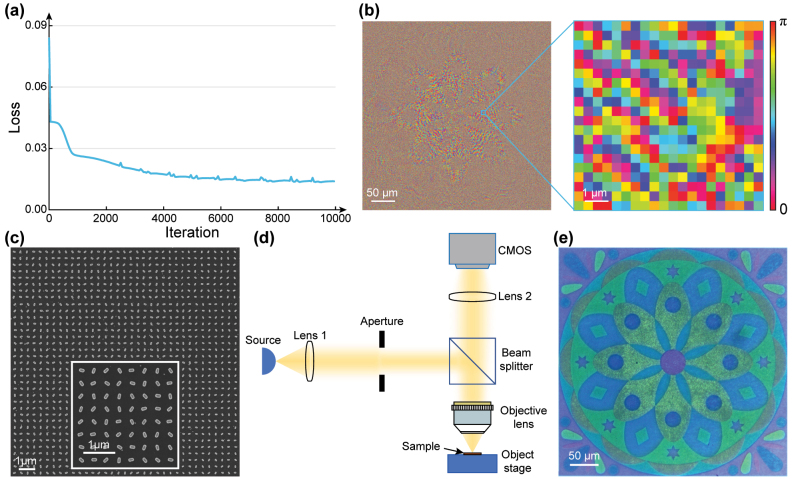
Optimized orientation angle distribution of the metasurface and nanoprinting image observation in Channel 1. (a) Loss versus number of iterations. (b) Optimized orientation angle distribution and its zoom-in view. (c) Partial SEM image of the metasurface sample. (d) Illustration of the reflection microscope optical path. (e) Experimentally captured nanoprinting image in the reflection mode.

To demonstrate the tri-channel metasurface, we designed a metasurface sample consisting of pixels of 1000 × 1,000, corresponding to geometric dimensions of 400 × 400 μm^2^. The target images for the three information channels are presented in Figure 2(c). The image in Channel 2 is designed for observation at a distance of 800 μm. The design wavelength of Channel 2 and Channel 3 holographic images is 550 nm, and the corresponding polarization state is LCP. For the two target holographic images, the weight coefficients *w*_1_ and *w*_2_ are set to 0.548 and 1, respectively. The maximum number of iterations is set to 10,000, and the loss function threshold is set to 0.01. The optimization of the orientation angle is performed in Python v3.10.11 utilizing the PyTorch v1.13.0 framework, with the Adam optimizer being employed. The variation of the loss function during the optimization iteration process is depicted in Figure 3(a) and (b) illustrates the optimized orientation angle distribution. The sample was meticulously fabricated using standard electron beam lithography (EBL) [40], [42], [43]. The partial scanning electron microscopy (SEM) images are displayed in Figure 3(c).

To observe the nanoprinting image encoded in Channel 1, an optical microscope (Motic BA310Met) equipped with a CMOS camera (Lbtek STC-MCS312POE) was employed. The metasurface was placed in the reflected light path, as illustrated in Figure 3(d). Figure 3(e) displays the image captured experimentally when the sample was illuminated with a white LED with a color temperature of 6,000 K. The captured image exhibits high resolution and clear details.

**Figure 4:**
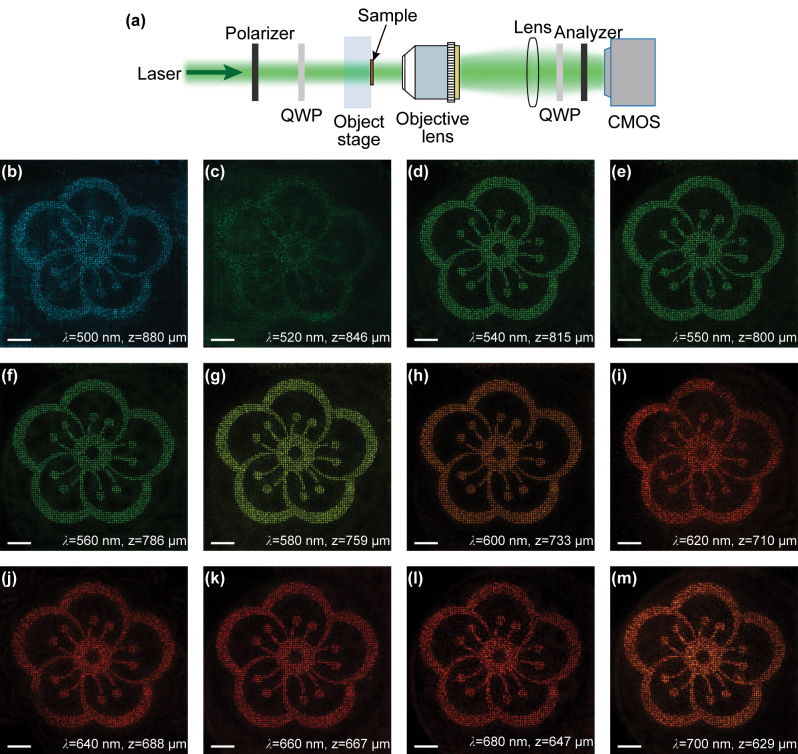
Observation of Fresnel holographic images in Channel 2. (a) Schematic diagram of the microscope optical path. (b–m) The experimentally captured holographic images at different wavelengths. All the scale bars are 50 μm.

The microscope light path, as illustrated in Figure 4(a), is employed to observe the Fresnel holographic image in Channel 2. To investigate broadband response of the metasurface, a super-continuum laser (YSL SC-pro) is utilized. The polarization state of the light waves is manipulated through the use of a polarizer and a quarter-wave plate (QWP), converting them into LCP light. Subsequently, the light waves sequentially pass through the metasurface and the microscope objective (50×, N.A. = 0.55). Before reaching the CMOS camera, another QWP and a bulky-optic analyzer are positioned to filter the LCP component of the transmitted light. According to the principle of Fresnel diffraction, the observation distance of the holographic images at different wavelengths is inversely proportional to the incident wavelength and this relationship can be approximately described by:

$$z_{1}=\frac{z_{0}\lambda_{0}}{\lambda_{1}}$$

where, *λ*_0_ and *z*_0_ are the designed wavelength and observation distance, respectively, while *z*_1_ represents the observation distance at the wavelength *λ*_1_. Figure 4(b)–(m) present the holographic images illuminated by a broadband laser source with wavelengths ranging from 500 nm to 700 nm in 20 nm increments, alongside the results for the designed wavelength of 550 nm. It’s evident that clear, high-fidelity images can be obtained near the design wavelength, but as the incident light wavelength deviates from the design value, image quality begins to slightly deteriorate.

**Figure 5:**
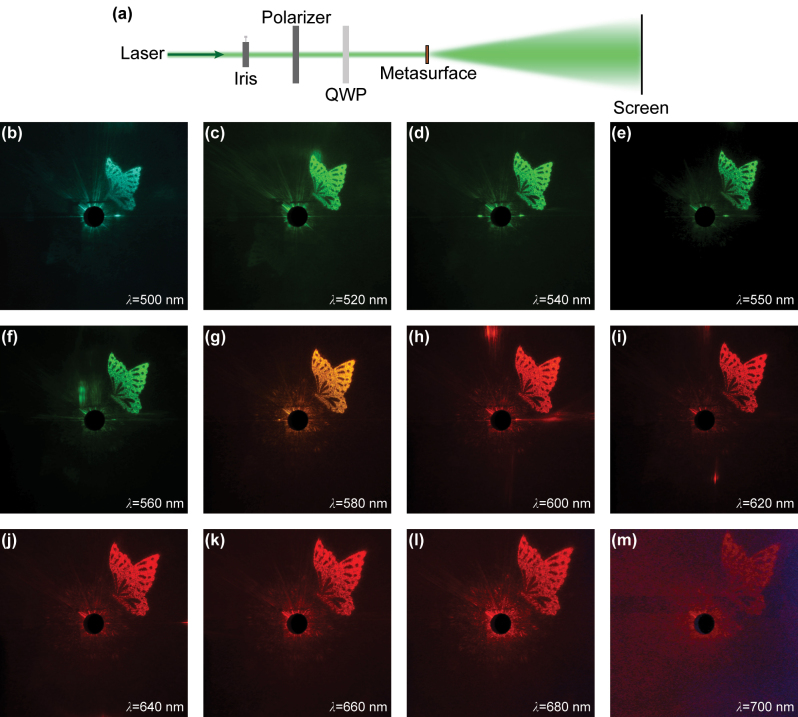
Observation of Fourier holographic images in Channel 3. (a) Schematic diagram of holographic optical path. (b–m) The experimentally captured holographic images under LCP light illumination at different wavelengths.

**Figure 6:**
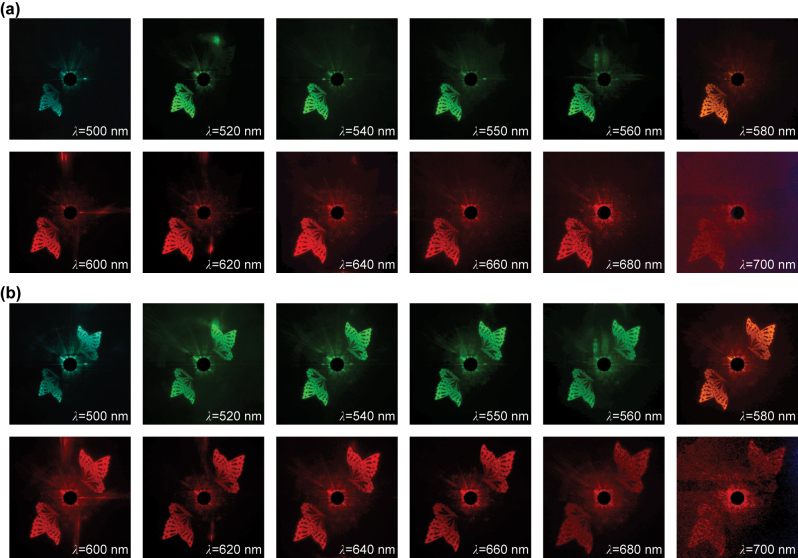
The experimentally captured holographic images in Channel 3 under (a) RCP and (b) LP light illumination at different wavelengths.

Utilizing the optical path depicted in Figure 5(a), the Fourier holographic images in Channel 3 are observed. The output light from the super-continuum laser is manipulated into specific polarization states with the assistance of the polarizer and QWP. Subsequently, the light passes through the metasurface and reaches a white screen positioned 15 cm away from metasurface, featuring a round aperture in the center. This aperture allows the zero-order light to pass through. A commercial camera (Nikon 5100) is used to capture the holographic images on the screen. As the wavelength varies from 500 nm to 700 nm, the holographic images under LCP light illumination are presented in Figure 5(b)–(m). Clearly, holographic images can be observed across a broad wavelength range, with the highest image quality attained at the design wavelength. Furthermore, holographic images under RCP and linearly polarized (LP) light illumination are shown in Figure 6(a) and (b), respectively. It is worth noting that when the helicity of the light changes, the position where the holographic image appears will change to a centrally symmetrical position because the geometric phase becomes the opposite number. As LP light can be regarded as the linear superposition of LCP and RCP light, the holographic images obtained with LP light are approximately an overlay of those obtained with LCP and RCP light. Importantly, with the propagation phase being independent of polarization state, a shift from LCP to RCP light results in a deviation from the optimized phase distribution, leading to a decrease in image quality at the design wavelength. Furthermore, the amplitude and propagation phase vary with wavelength, causing the image quality to gradually deteriorate with wavelength deviation, as shown in Figures 5 and 6.

**Figure 7:**
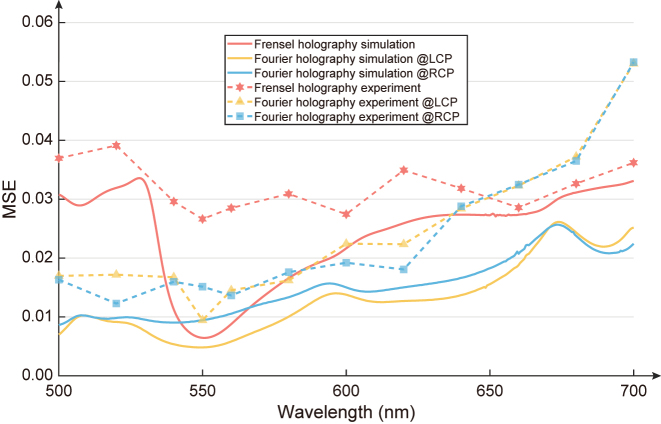
The MSE between the target images and the simulated results, as well as the experimentally captured images.

To quantitatively investigate the relationship between the quality of holographic images and wavelength, we calculate the MSE between the target images and the simulated results, as well as the MSE between the target images and the experimentally captured images. The solid lines and dotted lines in Figure 7 represent the results of simulations and experiments, respectively. The red curves, yellow curves, and blue curves correspond to the results of Fresnel holography under LCP light (Channel 2), Fourier holography under LCP light (Channel 3), and Fourier holography under RCP light. It is evident from the figure that at the design wavelength of 550 nm, Channels 2 and 3 achieve the highest image quality in both experiments and simulations. Additionally, as observed in Figure 7, there is a slight degradation in the image quality in the experimental results compared to the simulations. Several factors contribute to this disparity. Firstly, fabrication errors, which are inevitable, can lead to deviations in the complex amplitude of the metasurface from the designed results, introducing some background noise. Secondly, errors in optical path adjustments and variations in the polarization state can also introduce noise into the images. Furthermore, slight misalignments in image capture during the experiment can also influence the results of the MSE calculation. Nevertheless, it’s worth noting that the holographic images obtained experimentally still exhibit high image quality.

## 4 Discussion

The proposed tri-channel metasurface in the spatial domain offers several significant advantages. To begin with, it leverages three independent information channels to encode three distinct images within a single metasurface. This translates to an increase in information density when compared to previous single-functional metasurfaces. Furthermore, the spatial arrangement of these three information channels ensures they are well-separated in space. As a result, crosstalk between different channels is minimal. This feature is essential for delivering high-quality displays of three images without interference or overlap. In terms of design and fabrication, consisting of single-layered and single-celled nanostructures, the metasurface boast minimalist configurations. This simplicity in design offers an advantage in terms of scalability and easiness of fabrication. Additionally, the processing of these metasurfaces is compatible with mature semiconductor processing technologies. This compatibility makes large-scale processing and manufacturing convenient and cost-effective.

## 5 Conclusions

In summary, we propose and experimentally demonstrate a multifunctional metasurface for structural-color nanoprinting embedded with multi-domain spatial light field information. This metasurface effectively encodes three distinct images – namely, a color nanoprinting image, a Fresnel holographic image, and a Fourier holographic image – within a single metasurface. These images can be observed at spatially separated planes at different distances from the metasurface. Our experimental results confirm that all three images exhibit high quality and show virtually no inter-channel crosstalk. This tri-channel metasurface offers not only an increase in information density but also the assurance of high-quality, interference-free display of multiple images. Its minimalist design and compatibility with semiconductor processing technologies render it a promising candidate for a wide range of applications, from advanced displays to optical storage systems. As the field of metasurfaces continues to progress, these advancements hold great promise for the development of more compact, efficient, and versatile optical devices.
